# The neural basis of spatial vision losses in the dysfunctional visual system

**DOI:** 10.1038/s41598-017-11364-0

**Published:** 2017-09-12

**Authors:** Jinfeng Huang, Yifeng Zhou, Caiyuan Liu, Zhongjian Liu, Chunmeng Luan, Tzvetomir Tzvetanov

**Affiliations:** 1 Chinese Academy of Sciences Key Laboratory of Brain Function and Disease, School of Life Sciences, University of Science and Technology of China, Hefei, Anhui People’s Republic of China; 2 Research and Treatment Center of Amblyopia and Strabismus, University of Science and Technology of China, Hefei, Anhui People’s Republic of China; 3 Technical Research & Development Center, North Huajin Chemical Industries Group Corporation, Panjin, Liaoning People’s Republic of China; 4 School of Computer & Information, Hefei University of Technology, Hefei, Anhui 230009 People’s Republic of China

## Abstract

Human vision relies on correct information processing from the eye to various visual areas. Disturbances in the visual perception of simple features are believed to come from low-level network (e.g., V1) disruptions. In the present study, we modelled monocular losses in spatial vision through plausible multiple network modifications in early visual coding. We investigated perceptual deficits in anisometropic amblyopia and used the monocular tilt illusion as a probe of primary visual cortex orientation coding and inhibitory interactions. The psychophysical results showed that orientation misperception was higher in amblyopic eyes (AE) than in the fellow and neurotypical eyes and was correlated with the subject’s AE peak contrast sensitivity. The model fitted to the experimental results allowed to split these observations between different network characteristics by showing that these observations were explained by broader orientation tuning widths in AEs and stronger lateral inhibition in abnormal amblyopic system that had strong contrast sensitivity losses. Through psychophysics measures and computational modelling of V1, our study links multiple perceptual changes with localized modifications in the primary visual cortex.

## Introduction

Visual perception relies on correct processing from the eye, through the retina, LGN, primary visual cortex and up to higher visual areas that create the percept of our environment. Dysfunction of even one stage leads to visual losses that are detrimental to a person’s well-being. Some perceptual disruptions in spatial vision are associated with neural network changes in the striate cortex. The link between these multiple perceptual losses and the underlying network modifications is under intensive investigation and will provide important insights for plausible treatments.

One typical model of striate cortex modifications and low-level visual disturbances is amblyopia^1–4^, commonly known as lazy eye. It is a developmental disorder due to an abnormal visual experience during a critical period in early childhood, such as strabismus (a misalignment of the visual axes), anisometropia (strong refractive difference between the two eyes), and mixed and form deprivation (exclusion of all visual information other than light) amblyopia^5, 6^. It is characterized by impaired vision in the absence of overt pathology of the visual system that cannot be corrected by refractive means. Amblyopia affects 2–5% of the population^7–10^, and approximately half of individuals with lazy eye have anisometropic amblyopia^7^. Adult amblyopia is difficult to cure using currently available treatments. Because different kinds of amblyopia seem to show different underlying neural deficits^6^, here, we focus on anisometropic amblyopia as a model of neural dysfunction.

In behavioural research, anisometropic amblyopia showed deficits for higher cognitive functions, for instance, numerosity, reading, and perception of real-world scenes. Amblyopes suffer strong perceptual disturbances from low- to high-levels of visual processing such as orientation, motion, spatial position, global form perception, object recognition^3, 4, 11–13^, together with partial or complete loss of binocular function (3 D vision)^1, 6^. The most basic spatial vision deficits are measured through contrast sensitivity^6, 14–16^, vernier alignment thresholds^13^, orientation thresholds^17, 18^ and spatial interactions^19–21^, which are commonly associated with low-level feature coding and neural network disruption in primary visual cortex.

In neurophysiological research, it was found that neural impairments first appeared at V1^1, 14, 22, 23^. The retina and lateral geniculate nucleus (LGN) showed little significant abnormality in both anatomic and physiologic aspects^24–27^, while some studies, only recently with the help of fMRI measures, showed plausible functional and structural deficits at the thalamic level^28, 29^. Binocular functions also showed dramatic changes in area V1^22^ with the discovery of physiological and anatomical disruption of binocular organization^22, 30^. At the monocular level, many changes in neurons linked to the amblyopic eye (AE) were found, e.g., a reduced number of neurons responding to the AE^30, 31^ or neural under-representation of SF^1, 16^. On the other hand, there were no clear systematic neuronal contrast sensitivity changes^4, 30, 31^ and no clear neuronal evidence for receptive-field jitter^1^. There was no clear link between the behavioural contrast sensitivity losses and its assumed neurophysiological substrate – the neuronal contrast sensitivity.

To reveal plausible early mechanisms of amblyopia dysfunction, behavioural studies investigated lateral interactions in purportedly striate cortical networks. Specifically, they investigated lateral interactions in the amblyopic striate cortex monocularly, since V1 is largely accepted as the main site for mediating the lateral masking phenomena observed behaviourally^32–35^ and its basis may be the long-range horizontal intrinsic connections that have been observed in visual cortex^36^. Polat & Sagi and their collaborators showed reduced lateral excitatory interactions in anisometropic amblyopia^20, 37, 38^, which was also reported in other results^39, 40^. Further abnormalities in the interactions were also found for second-order stimuli, lateral interactions and texture patterns^41, 42^.

However, overall, a direct relationship between the modifications in the amblyopic striate cortex at the monocular level, i.e., its neural bases, and the most obvious monocular perceptual deficits in processing simple features in AEs is lacking and remains poorly understood^22^. Our study applies a simple neurophysiological model of visual perception and links multiple perceptual deficits in anisometropic amblyopia to localized neuronal network changes in V1.

Here, we used the centre-surround tilt illusion^43–45^, where an inducing line or grating at one orientation affects the perception of a simultaneously presented test line or grating at a different orientation, to access local and lateral (centre-surround) inhibitory interactions. A simplified model of V1 about centre-surround interactions e.g., ref. 44 was instantiated, implementing a theoretical population of neurons performing local orientation processing (orientation hypercolumn) and the interactions between lateral hypercolumns. It is shown that the model allows one to infer theoretical parameters of local orientation tuning widths and lateral inhibitory strength within the primary visual cortex from the direct tilt repulsion curve^46, 47^. It allows the further prediction of the contrast sensitivity function (CSF) of subjects by including contrast and spatial frequency coding and thus extracts an underlying theoretical neuronal sensitivity function.

Our experimental results show that the tilt misperception in AEs is higher than that in non-amblyopic eyes (NAEs) and neurotypical eyes (NTEs). This orientation misperception is highly correlated with the contrast sensitivity in AEs at peak SF. These phenomena could be explained at V1 neuronal network changes through wider orientation tuning widths in AEs and increased centre-surround inhibition in the neuronal system of AEs with stronger contrast sensitivity losses. Thus, our study provides new insights into the relationship between perceptual losses, visual misperceptions and their neuronal substrate modifications at early coding stages.

## Results

### Does an eye’s physical state influence contrast sensitivity?

**Figure 1:**
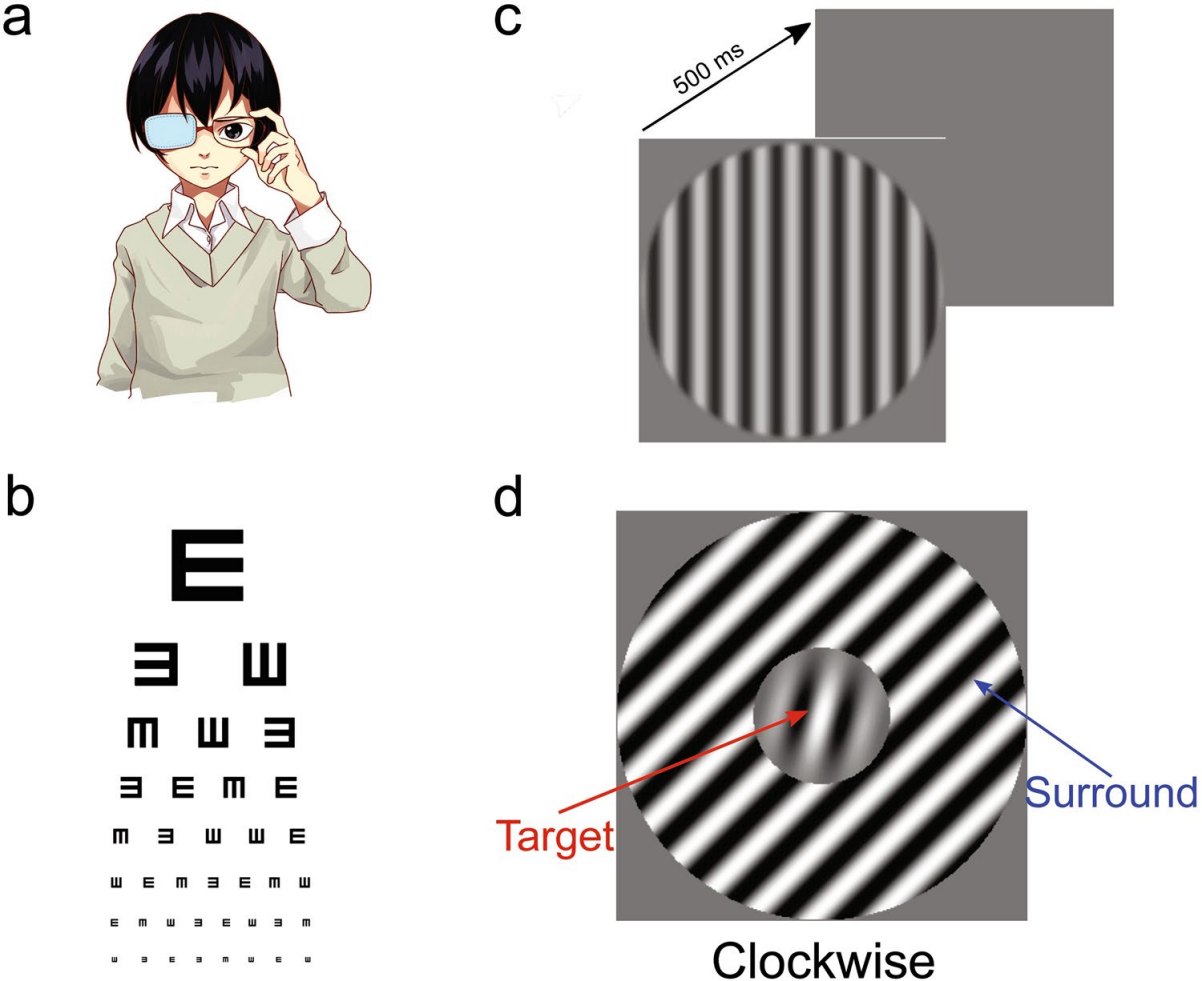
Schematic Diagram of the Experiment. (**a**) A cartoon example of a person who is doing the experiment (drawn by one of the authors). (**b**) Illustration of visual acuity chart. (**c**) Example of stimuli for CSF measure. Each trial has two intervals and there is a 500 ms blank between them. The stimulus will randomly appear in either of them. (**d**) Example of stimuli for tilt illusion measure. In this stimulus, the orientation of the surround is 30 degrees, and the target is clockwise to the vertical.

**Figure 2:**
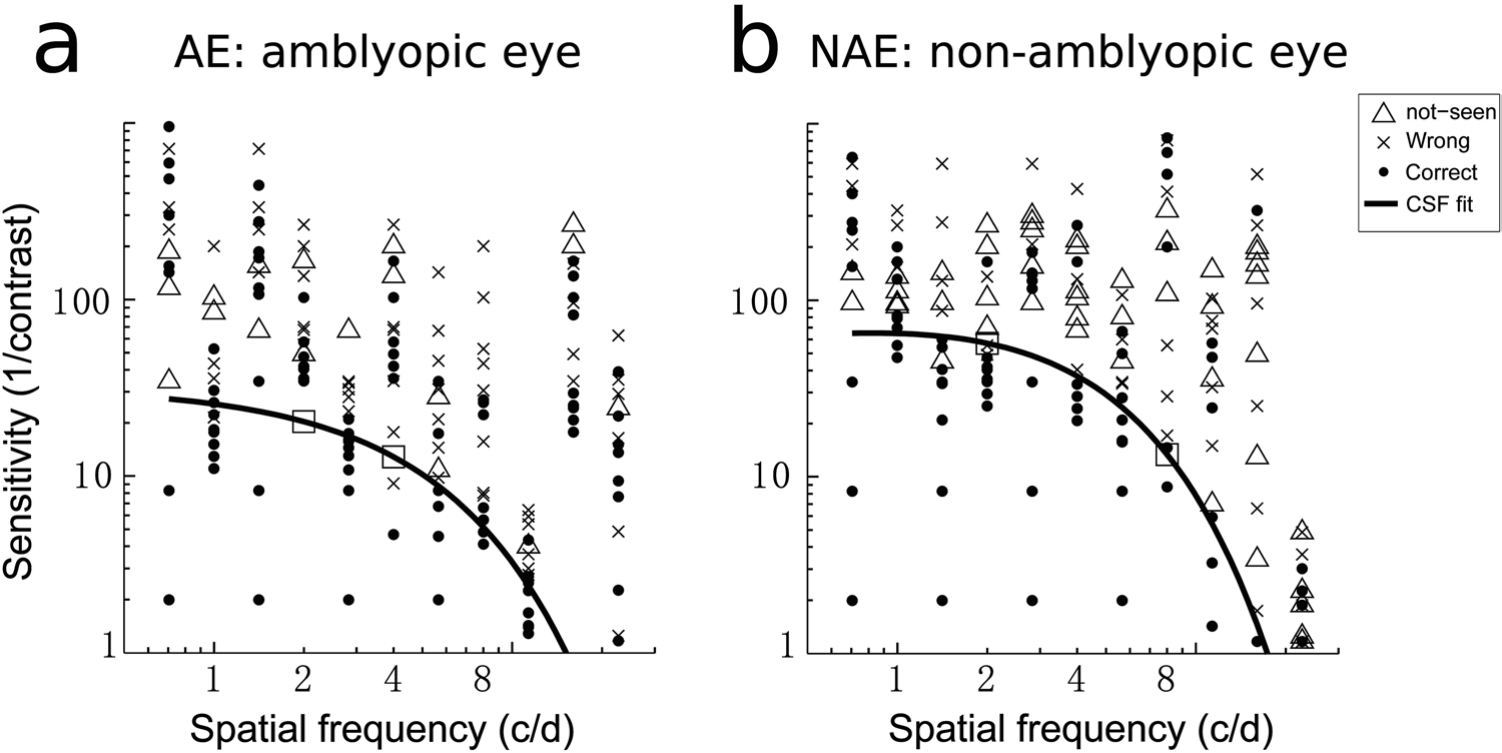
Examples of CSF Measures and Fits Results.Examples of CSF measures and fits results in the unforced 3-responses design for AE (**a**) and NAE (**b**). The two open squares depict the chosen SFs for tilt measures.

**Figure 3:**
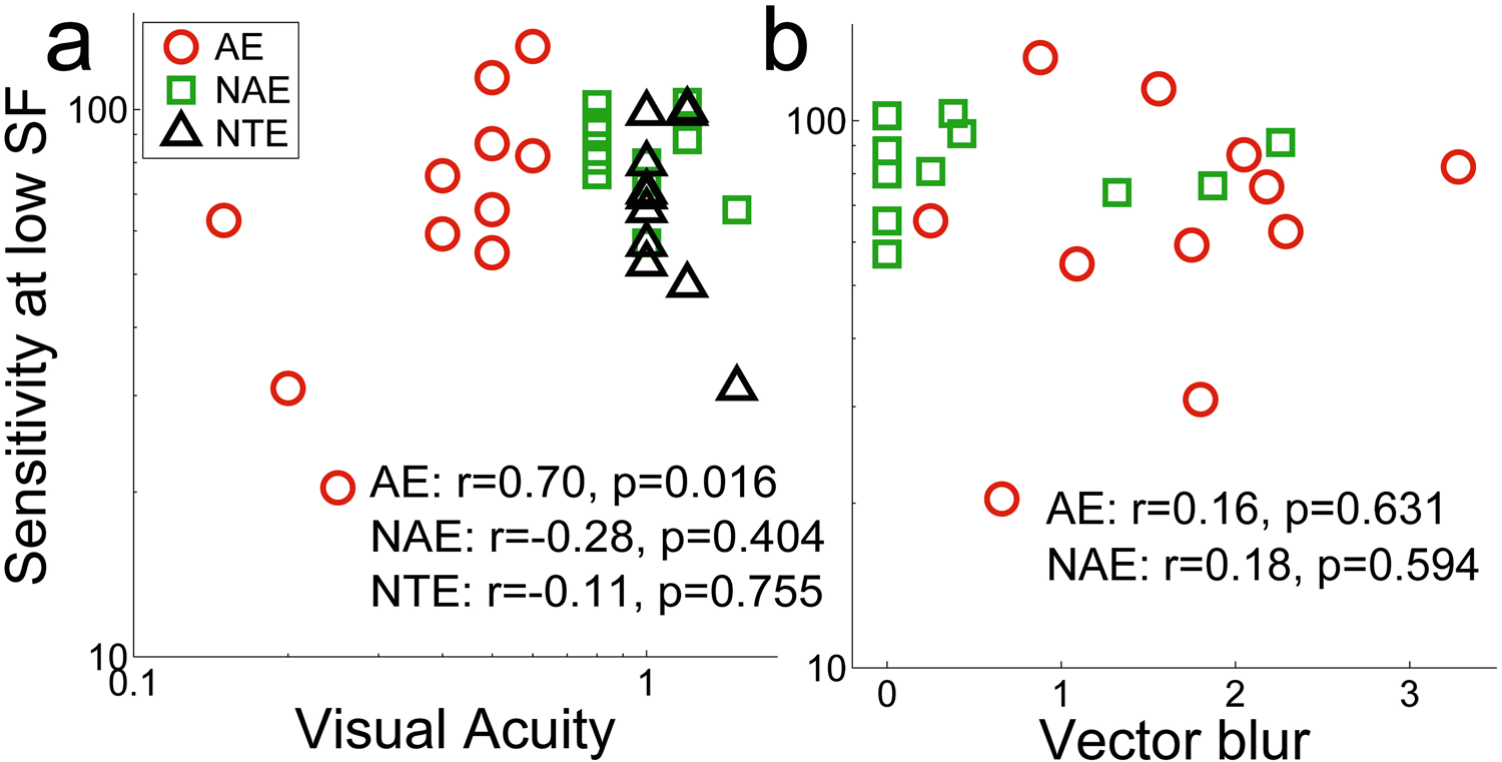
Sensitivity vs. VA or Vector Blur. Sensitivity at low SF vs. decimal visual acuity (**a**) or vs. Vector blur (**b**). (r and p are Spearman rank correlations and probability; p < 0.05 is considered significant).  represents AE;  represents NAE; △ represents NTE.

First, we measured the individual monocular visual acuity (VA) and CSF of anisometropic amblyopes for AE and NAE and of neurotypical subjects’ eyes (Figs 1b,c and 2). Figure 3a presents the VA for each eye versus the contrast sensitivity measured at a lower SF. VA in the AEs was systematically lower than that in the NAEs (paired t-test: t(10) = 5.73, p = 0.00019) and NTEs (unpaired t-test: t(20) = −10.1, p = 2.7 * 10^−9^). VA of a given eye correlated with low SF sensitivity in the AEs but not in the NAEs and NTEs. By contrast, vector blur^2^, which relates to the optics of the eye (see Materials and Methods), did not correlate with contrast sensitivity (Fig. 3b), while it was different between AEs and NAEs (t (10) = −2.55, p = 0.029). Thus, the physical differences of the eye were unrelated to our subjects’ perceptual sensitivity changes.

### Tilt repulsion

**Figure 4:**
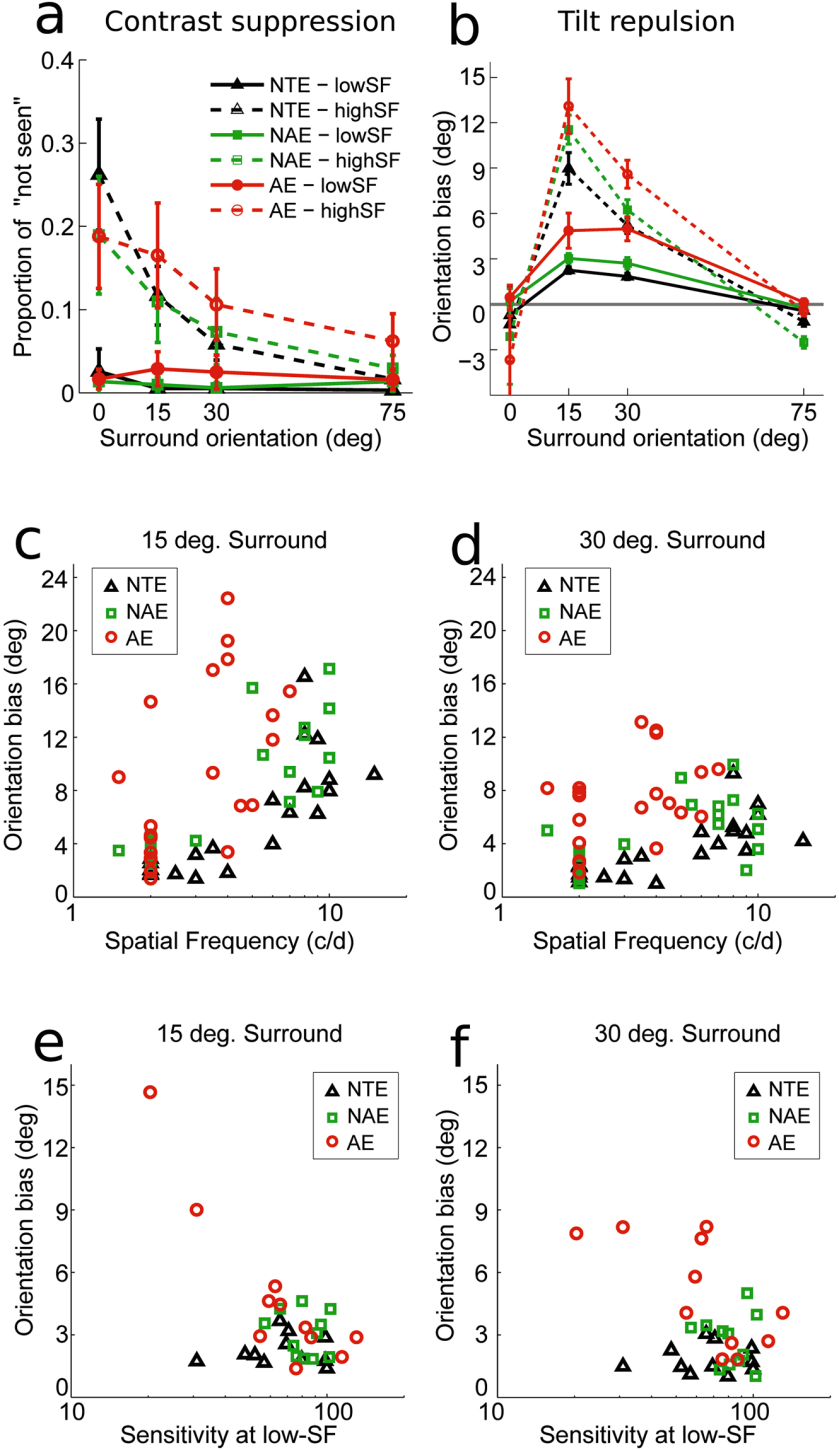
CSF and Tilt Illusion Amplitudes Co-vary in Amblyopic Eyes. (**a**,**b**) Orientation perception results, with (**a**) suppression effects measured through proportion of “not seen” and (**b**) tilt repulsion results as a function of surround orientation and SF of the stimuli for each type of eye. Solid and dashed lines depict low and high SF respectively. (**c**,**d**) Correlations between tilt repulsion and all measured SFs for each type of eyes, for surround orientations of (**c**) ±15 degrees and (**d**) ±30 degrees. (**e**,**f**) Correlation between tilt repulsion (bias) and contrast sensitivity at the lowest measured SF for surround orientations of (**e**) ±15 degrees and (**f**) ±30 degrees.  represents AE;  represents NAE; △ represents NTE.

From the CSFs, we chose two SFs, one near the peak sensitivity (low-SF) and one higher (high-SF) (Fig. 2, squares), at which to measure the individual tilt repulsion effect, with the condition that the sensitivity at the high SF was high enough to allow for stimulus perception at the next measures of centre-surround tilt misperception. Then, we measured each subject’s amount of orientation misperception (tilt illusion) at each SF for centre-surround orientation differences of 0, ±15, ±30 and ±75 degrees (stimulus example in Fig. 1d; the chosen SF for each amblyopic participant in the tilt illusion measure are shown in Table 1). The subjects were instructed to respond to the orientation only if they saw an oriented stimulus in the centre; otherwise, they were to hit a third key for “not seen” (e.g., noisy or greyish centre) (Fig. 1d). This provided data related only to clear orientation perception; that is, the measure of misperception was independent from centre-surround contrast suppression effects (Fig. 4a).

**Table 1:** SF sizes of each amblyopic participant in the tilt illusion measure.

Subject	Sex	Age	AE (SF, c/d)		NAE (SF, c/d)	
			Low	High	Low	High
1	M	26	2	4	2	9
2	M	26	2	4.5	2	10
3	M	23	2	3.5	2	10
4	M	24	2	6	2	10
5	M	23	2	4	2	7
6	F	24	2	4	2	8
7	F	26	1.5	3.5	2	7
8	F	29	2	4	2	5.5
9	M	21	2	5	3	8
10	M	25	2	7	2	7
11	M	25	2	6	1.5	5
12	M	26	2	—	2	4
13	M	25	1.5	—	1.5	9
14	M	28	2	4.5	2	6

First, our results show that our indirect measure of the surround suppression effect on perception was found to be dependent on SF as well as surround orientation (SO), while factor Eye had a tendency of an effect only within amblyopic subjects (within-subject design). We also found a strong interaction between SO and SF (ANOVAs performed on the SO sign-pooled values (4 levels) with logit-transformed proportions, setting any zero value to 1/120; see Fig. 4a and Supplementary Table S1). Thus, the parallel surround configuration had a strong detrimental effect on the detectability of the centre target despite its nearly full contrast. Therefore, it automatically decreased the number of trials available for the correct estimation of orientation perception, especially at high SFs, for the iso-orientation configuration (Fig. 4a). Additionally, we do not discuss further SOs of 75 deg. that correspond to the indirect tilt effect attributed to higher levels of visual processing^46–48^ and about which the V1 model proposed here cannot make predictions. As a consequence, we restricted our data analyses to SO of 15 and 30 degrees corresponding to the direct repulsion effect.

The orientation discrimination thresholds (or spread of psychometric function, σ in equation (4)) were strongly modulated by all factors of Eye, SO, and SF between NTE and AE or NAE but not between AE and NAE. There was also a main interaction between SO and SF across all eye comparisons showing a different trend of threshold variation with SO at different SFs (for details, see Supplementary Table S2), which confirms previous reports of SF-specific AE threshold elevations^17, 49^.

Perception through AE exhibited much stronger tilt repulsion effects at ±15 and ±30 degrees SOs across all measured SFs in comparison to the NAE and NTE, stronger tilt illusion at higher SFs, and significant interactions between SO and SF across all eye comparisons (Fig. 4b–d and Supplementary Table S3). Thus, we observed the standard tilt repulsion effect at SOs of ±15 and ±30 degrees together with the increase of repulsion at higher SFs. Figure 4c,d re-plot individual biases at each measured SF, showing the general trend across eyes of higher tilt repulsion with increasing SFs^45, 50^ (Linear regression for bias: SO = 15 deg., AE: r^2^ = 0.29, p = 0.01; NAE: r^2^ = 0.71, p = <0.0001; NTE: r^2^ = 0.58, p = <0.0001. For SO = 30 deg., AE: r^2^ = 0.18, p = 0.051; NAE: r^2^ = 0.29, p = 0.010; NTE: r^2^ = 0.51, p = 0.0002).

### CSF and Tilt Illusion Amplitudes Co-vary in Amblyopic Eyes

Importantly, the amount of tilt illusion at low SFs was found to be negatively correlated with the contrast sensitivity at low SF in AE, while there were no relations for NAE and NTE (Fig. 4e and f, AE: at θ = 15 deg., r = −0.77, p = 0.008, at θ = 30 deg., r = −0.59, p = 0.06; NAE: at θ = 15 deg., r = −0.21, p = 0.54, at θ = 30 deg., r = −0.01, p = 0.99; NTE: at θ = 15 deg., r = −0.03, p = 0.95, at θ = 30 deg., r = −0.05, p = 0.90). Specifically, near the optimal SF in the AEs, stronger orientation misperception due to the surround was accompanied with lower contrast sensitivity.

To confirm that the finding at low SF is a general phenomenon for anisometropic amblyopes, we collected additional and all available data at low SF from anisometropic amblyopes (thus increasing subjects’ number from 11 to 21; see Supplementary Material, Supplementary Table S4, Supplementary Fig. S1 and Materials and Methods for further information). Thus, a total of 21 anisometropic amblyopes took part in the experiment at low SF. The results confirmed the new anti-correlation finding in AE (AE: at θ = 15 deg., r = −0.60, p = 0.0037, at θ = 30 deg., r = −0.64, p = 0.0017; NAE: at θ = 15 deg., r = −0.22, p = 0.34, at θ = 30 deg., r = −0.078, p = 0.74; Supplementary Fig. S2).

### Neurophysiologically Based Model Of Perception

The correlation between these two independent measures hinted towards a common explanatory source. We considered that these correlations might stem from the fact that both types of stimuli cover an amount of the central visual field that encompasses multiple non-overlapping receptive fields of neurons. Thus, we hypothesized that our behavioural results are also strongly shaped by known inhibitory lateral interactions in the primary visual cortex, which is the visual area considered to be the substrate of tilt misperception and contrast detection^45, 51, 52^. Although very appealing, psychophysical modelling based on neuronal responses of primary visual cortex relates each variable to different tuning characteristics.

**Figure 5:**
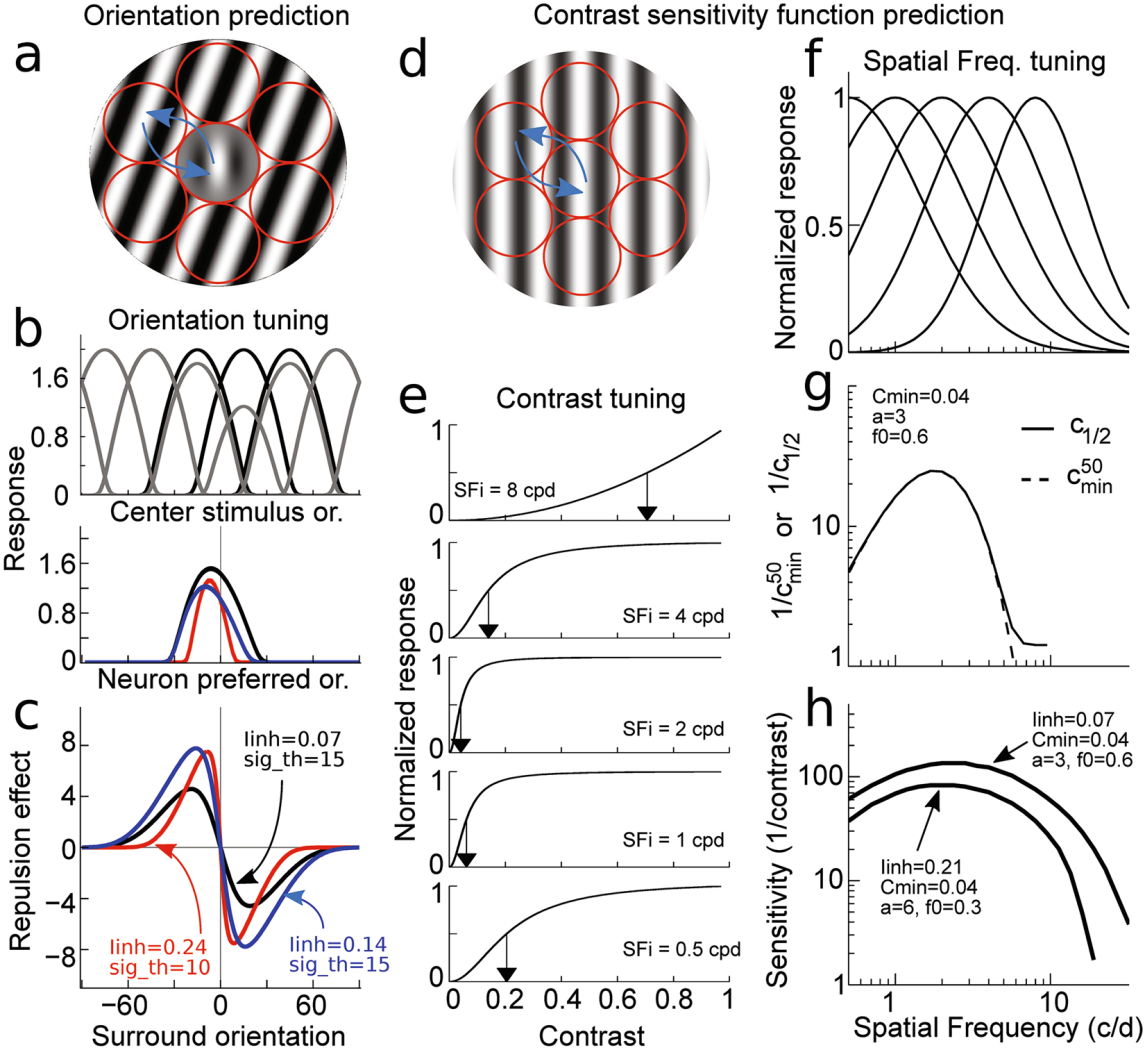
V1 Model Illustration. (**a**–**c**) Example and prediction for orientation coding and decoding. (**a**) Illustration of the center-surround stimulus and putative orientation hypercolumns (red circles, ) paving it together with their lateral interactions (blue arrows, shown for only one pair of hypercolumns) (**b**) Top: Sample of orientation tuning curves in the model when no surround is present (solid black) and when a surround of +15 degrees is present (solid grey). Bottom: Response of the neuronal population to centre of 0 deg. and surround orientation of +15 degrees. (**c**) Orientation prediction of the model from the population responses for various surround orientations. In (**b**) and (**c**), the red, black and blue lines are corresponding to the three different set of parameters in the same color which were shown in (**c**). (**d**–**h**) Example and prediction for SF and contrast tuning coding and decoding. (**d**) Illustration of a low-contrast cosine grating stimulus and its paving by orientation hypercolumns (red circles, ) and their lateral interactions (blue arrows). (**e**) Examples of contrast response functions in the model at few preferred SFs. Arrows depict *c*_*1/2*_ where response is at half of neuron’s maximum response. (**f**) SF tuning examples, with the characteristic tuning width decrease with increasing preferred SF. (**g**) Example of the relation between *c*_*min*_, best semi-saturation constant at a given SF, and preferred SF of the neuron that follows a bell shaped function. (**h**) Examples of CSF prediction for two sets of model parameters.

Orientation misperception in the centre-surround paradigm is modelled through orientation tuning and lateral inhibitory interactions^44, 51, 53, 54^ between non-overlapping orientation tuned neurons (Fig. 5a–c). Based on the orientation hypercolumns of Hubel & Wiesel^55^, it is assumed that the centre target stimulus activates a single hypercolumn population of neurons while the surrounding grating activates hypercolumns in the closest vicinity (Fig. 5a). These hypercolumns interact through lateral inhibitory interactions (see Materials and Methods) that can be considered a substrate of the centre-surround receptive-field structure^55^, and the perceived orientation of the target is obtained by vector average decoding of the central hypercolumn activities^56^. The presence of a surround globally decreases the maximum firing rate of neurons with preferred orientations close to the surround value and thus changes the profile of neuronal population activity in a non-linear manner (Fig. 5b). In this model, two major parameters affect the final tilt illusion: the local orientation tuning width (*σ*_*θ*_) of the hypercolumn of neurons (Fig. 5b) and the strength of the lateral interactions between orientation hypercolumns (*I*_*inh*_; blue arrows in Fig. 5a). The tuning width (*σ*_*θ*_) globally affects the shape of the tilt illusion curve (its peak position and amplitude; in Fig. 5c, compare the red curve to the blue and black curves), while *I*_*inh*_ mainly impact the amplitude of tilt misperception (compare the black and blue curves in Fig. 5c). Thus, from the centre-surround tilt misperception measures, one can infer these two theoretical population characteristics (see Materials and Methods for details).

On the other hand, perception of contrast at various SFs, the contrast sensitivity function (CSF), is thought to arise from the SF and contrast tuning properties of V1 neurons^30, 31, 57–60^ (Fig. 5d–g). To measure the CSF, since the grating is spatially extended, the strength of lateral interactions should also play a role in contrast detection (Fig. 5d). Thus, for fixed *I*_*inh*_, if the SFs and contrast tuning relations are known (Fig. 5e–g), that is, one assumes contrast detection is based on the best contrast tuning function with the smallest semi-saturation constant (*c*_*min*_, Fig. 5e) and its variation across SFs is parameterized (Fig. 5g), one can infer the CSF from standard Signal Detection Theory (Fig. 5h, see Materials and Methods for further details) and link behavioural and neural contrast sensitivities. For a fixed CSF model, because stronger inhibition is known to increase the contrast threshold of neuronal firing^61^, the main expected effect of *I*_*inh*_ is that a stronger value should decrease the CSF and a weaker one should increase it (example in Fig. 5h).

In this model, the tilt illusion and CSF (Fig. 5c and h) are related through a single parameter - the amount of surround-to-centre inhibition (*I*_*inh*_). On one side, the amplitude and shape of orientation misperception are dependent on centre-surround inhibition and orientation tuning width (Fig. 5a–c), while the CSF is dependent on lateral inhibition, together with the smallest contrast semi-saturation constant (*c*_*min*_) and its relation to the SF tuning (Fig. 5d–h) (see Materials and Methods for model details).

**Figure 6:**
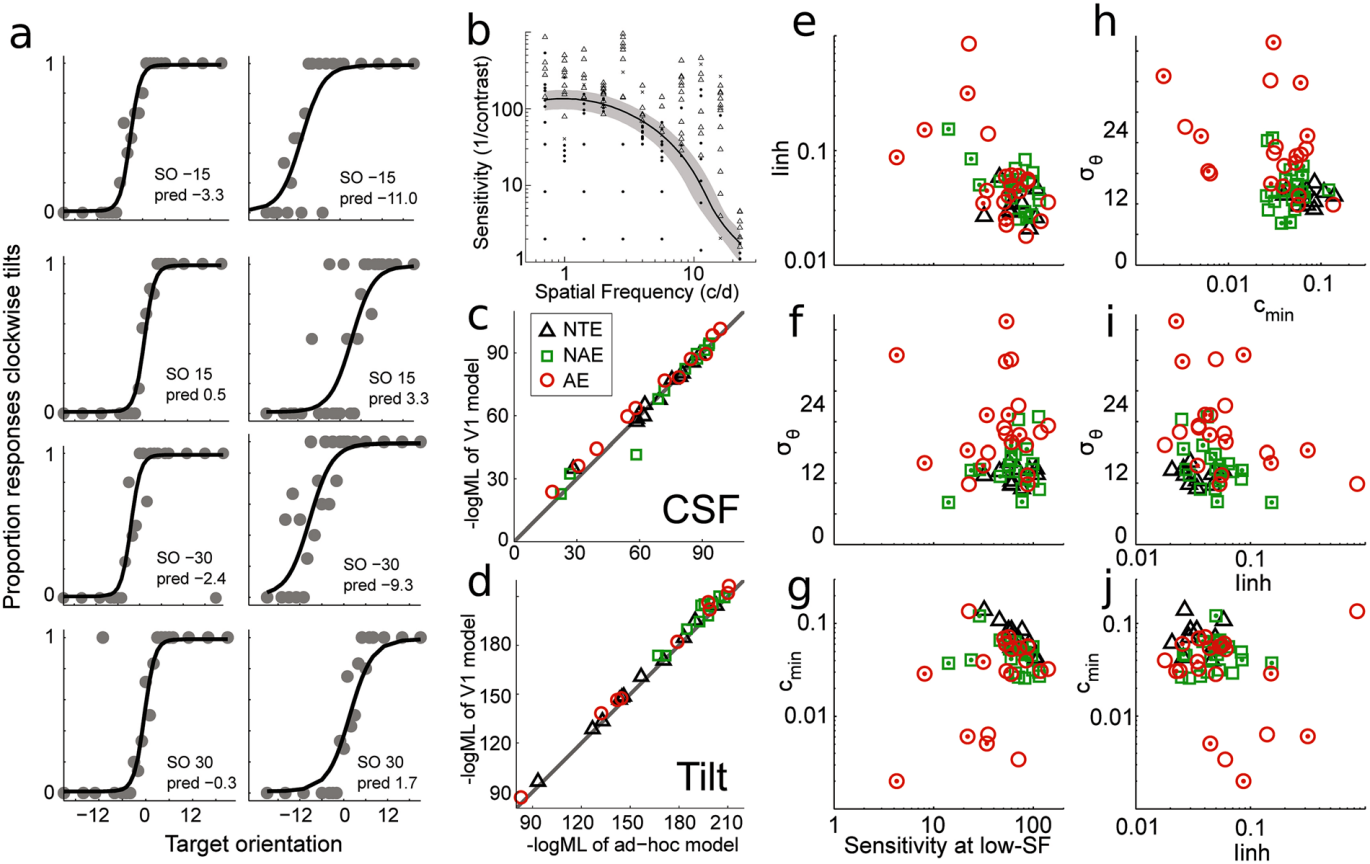
V1 Model Fit Results(**a**) Psychometric functions data (• = response means at the given target orientation) and fits (black solid lines) of orientation discrimination data. Each panel correspond to one surround orientation (SO) and also displays the predicted perceived vertical. (**b**) CSF data (•, × and △; see Fig. 3 for details) and fit (solid line). Grey area depicts the 16% to 84% psychometric function spread around the midpoint (black curve). (**c**,**d**) Comparison of Maximum Likelihood (−log(ML)) values of ad-hoc *vs*. V1 model fits for CSF (**c**) and Tilt (**d**) data separately. (**e**–**g**) Model fit results for the three main parameters of interest (**e**) *I*_*inh*_, (**f**) *σ*_*θ*_, and (**g**) *c*_*min*_, for each type of eye, as a function of model predicted sensitivity at low-SF. (**h**–**j**) Correlations between the three parameters: *I*_*inh*_, *σ*_*θ*_, and *c*_*min*_.  represents AE;  represents NAE; **△** represents NTE. Each symbol with a dot in the center is from the 10 additional subjects.

We fitted the model to each subject’s results as follows: for the tilt perception data to extract (*I*_*inh*_, *σ*_*θ*_) and then with fixed *I*_*inh*_ to the CSF data (to extract three parameters: *c*_*min*_, *a*, *b* –see Experimental Procedures for details; with *c*_*min*_ corresponding to the best neuronal contrast sensitivity across all SFs; see Fig. 5g, peak value). Figure 6a,b depict example fits of psychometric functions to the tilt perception and CSF data of one subject (amblyopic subject 1, NAE). First, across subjects, the fits provided by the physiological model were similar in quality to those provided by the standard ad-hoc psychometric functions (comparison of models predicted ML values, Fig. 6c and d), without strong differences in fit quality among the three types of eyes. Nevertheless, there were two small differences: (i) for the tilt data, across subjects, the model tended to be slightly worse than the ad-hoc 1D psychometric functions, but notwithstanding this, as in previous work, it provided a good explanation of the tilt misperception data^51^; (ii) for the CSF data, the V1 model prediction deviated more from the ad-hoc model with a decreasing −log(ML) value. This second effect is due to the particularity of the V1 model contrast response functions (CRFs) parameters relations, which, with lower CSF peak sensitivities, increase the spread (i.e., grey area in Fig. 6b, corresponding to shallower functions in the contrast dimension) while still catching the CSF shape in the SF dimension. Since the model still provides an overall good explanation of the main effect of peak decrease, we did not further investigate different or other relations between CRFs or SFs tunings.

The tilt data provided surround to centre inhibition strengths (Fig. 6e) together with orientation tuning width estimates (Fig. 6f). At low SFs, near the peak of contrast sensitivity, lateral inhibition in the AEs was globally similar to the two other types of eyes (Fig. 6e; AE vs. NAE: t(20) = 1.289, p = 0.212; AE vs. NTE: t(20.321) = 1.708, p = 0.103. On the other hand, *I*_*inh*_ within AEs and NAEs correlated with the eye’s contrast sensitivity (respectively, r = −0.500, p = 0.021; r = −0.451, p = 0.040) but not within NTEs (r = −0.13, p = 0.70). Orientation tuning widths were found to be much broader in AEs compared to NAEs or NTEs (respectively, t(20) = 5.010, p = 0.000067; t(22.530) = 4.925, p = 0.000059; Fig. 6f), but did not correlate with contrast sensitivity at the low-SF (AE: r = 0.006, p = 0.980; NAE: r = 0.040, p = 0.865; NTE: r = 0.09, p = 0.79). The CSF data, in combination with the tilt data fits (*I*_*inh*_), provided an estimate of the best neuronal contrast sensitivity (*c*_*min*_) of the subjects (Fig. 6g). This parameter was found to be different between NTEs and AE or NAEs (respectively, t(30) = −3.101, p = 0.004; t(30) = −3.157, p = 0.004), while it did not seem to differ between AEs and NAEs (t(20) = −0.951, p = 0.353). Notably, this parameter explained the differences between the various perceptual CSF measures among subjects with NTEs (r = −0.870, p = 0.00050) but not within NAEs or AEs (respectively, r = −0.411, p = 0.064; r = 0.224, p = 0.330). Thus, our data showed that *c*_*min*_ provided a good explanation of the peak sensitivity differences across subjects with NTEs without any relation to *I*_*inh*_, while within both eyes of amblyopes’ sensitivity changes were explained by increased *I*_*inh*_ without relation to *c*_*min*_. In addition, our tilt data was explained with a broader orientation tuning width of AEs than NAEs and NTEs without any relation to subjects’ CSF.

The above results showed that the observed correlations between the two behavioural measures (Fig. 4e,f) were differentially split between the different network characteristics (Fig. 6e–g). To further investigate this dissociation, we analysed all pairwise correlations between the three major parameters. Only NAEs’ correlation of orientation tuning width and lateral inhibition was significant, showing that the above dissociations were not due to a systematic co-variation of parameters across eye types (see Fig. 6h,i for details and Supplementary Table S5).

## Discussion

Our behavioural results and computational modelling are the first to link low-level perceptual deficits in the dysfunctional visual system, measured through visual contrast sensitivity and centre-surround tilt repulsion, to multiple changes at the monocular neuronal level. We used anisometropic amblyopia as a model of neuronal disruption of vision and found that lazy eyes exhibited wider orientation tuning widths and that generally both eyes of anisometropic amblyopes showed stronger lateral inhibition within eyes that have stronger contrast sensitivity deficits.

The contrast sensitivity function is one of the most basic psychophysical measures that is widely used in research and is also found in practical ophthalmic situations for characterizing low-level visual deficits^6, 62^. It is largely reported that amblyopic individuals can have weak to strong visual deficits by various low-level psychophysical measures in their AEs that can or cannot correlate with their acuity and sensitivity losses^6^. Nonetheless, neurophysiological reports about neuronal contrast sensitivities are at odds. Kiorpes and colleagues^30^ conducted both behavioural testing and electrophysiological recording on monkeys. They found significant peak contrast sensitivity losses in the anisometropic eyes of their monkeys during the behavioural test; however, in the subsequent physiological experiments, no reliable differences were found in neuronal contrast sensitivity between the two eyes in their V1 neuronal samples. Our study provides an explanation for this puzzling finding. Their behavioural deficits could stem from the stronger lateral inhibition in the amblyopic system, which was not accessible in their study. Our model results showed that the two eyes of our amblyopic subjects displayed globally similar neuronal contrast sensitivities, thus providing an interesting counterpart to the observations of Kiorpes *et al*.^30^.

Lateral interactions in low-level visual processing have been related to specific structures and connectivity within early visual areas^63^. Psychophysical studies have demonstrated the presence of abnormal spatial interactions in anisometropic amblyopes e.g., refs 19–21, 38, 39, 64, with the idea that some psychophysical measures directly probe inhibitory/excitatory lateral interaction connections^4, 20, 38, 41^. Our use of the centre-surround tilt illusion, which was interpreted almost 50 years ago as one instantiation of lateral inhibitory interactions between orientation sensitive neurons^45, 65^, as a probe was based on cumulative knowledge from physiology, behaviour and computational work that permitted modelling the supposed underlying computational structure creating the effect^44, 50, 51^. This allowed dissociation of the behavioural correlations between CSF and tilt illusion to specific network characteristics of orientation tuning widths, strengths of lateral inhibition, and neural contrast response functions. Our main findings of larger orientation tuning widths in the AEs, globally similar neural contrast functions between AEs and NAEs, and stronger lateral inhibition for both eyes in amblyopes showing stronger CSF losses demonstrate the necessity to have a multidimensional approach to visual neural dysfunctions.

The finding of broader orientation tuning widths in AEs resonates well with the results of other studies^25, 66, 67^. For instance, Faulkner *et al*.^66^ found that orientation tuning of cells dominated by the originally deprived eyes is significantly wider than that of cells dominated by the fellow eyes in kittens. Furthermore, the response rates or the variability of firing of neurons in the kittens did not have any obvious difference between the groups that could not have contributed to the tuning width difference, which is consistent with our modelling results.

Multiple authors have written about the astonishing illusory percepts reported by amblyopes through their weak eye in normal viewing conditions of static grating stimuli^68–70^, which demonstrates to the investigators and the patients the underlying neuronal disruption of the visual system. Our results are in line with the above work, providing a link between the perceptual outcome, misperception, and its substrate. There is more work to be done; for example, the centre-surround stimulus in our study is too short to change the subject’s perception of the stimulus taking place. The recent proposition of only a few categories of static grating illusory perception in the AE of amblyopes^70^ opens new perspectives and possibilities to further model the underlying dynamical changes in the abnormal neuronal system. In addition, our work investigated first-order tilt illusion, but there is evidence that second-order misperception in amblyopia is also abnormal^42^, whose neurophysiological modelling should further extend our understanding of the link between perception and neural network disturbances at a higher level of sensory processing.

It is known that both monocular and binocular deficits are found in anisometropic amblyopes. The underlying alterations in circuitry remain poorly understood^71^. Some researchers attribute this damaged neuronal circuitry to binocular suppression^72^, with the fellow eye suppressing the AE processing capacities. This line of thought is also supported by the findings that there seems to be a stronger contrast gain control from the fellow eye to both (1) the signal in the AE and (2) the contrast gain control signal from the AE^73, 74^. By contrast, our results show that affected amblyopes’ eyes exhibiting stronger lateral inhibition could occur at a monocular level before the fellow eye acts on the AE (see also the discussion of Bonneh *et al*.^38^ for such a proposition among other explanations). Therefore, another possibility can be put forward that the observed binocular suppression could naturally follow from monocular inhibition without any assumption of asymmetric binocular interactions^74^.

Interestingly, a close neurophysiological counterpart was recently reported in the strabismic visual system^71^. In this work, the authors showed that the monocular deficit was present before the binocular combination. For example, the simple cells in the strabismic cats exhibited large binocularity losses that were attributed to increased synaptic inhibition. Because simple cells receive direct input from the LGN and are primarily located in layer 4 of V1, they can reflect early neuronal processing of the AE. Furthermore, they established a circuit model and found that they can account for the observed damage only with plasticity at thalamocortical synapses. This interesting match between our study in anisometropic amblyopes and their results in strabismic animals strongly supports our interpretation but also provides a new line of research and understanding of the underlying neural system modifications due to abnormal visual experience and increased inhibition in the abnormal visual system^75–77^.

The above behavioural and modelling approaches are applicable to other conditions where persons exhibit changes in their visual abilities, whether pathological (e.g., schizophrenia, strabismus, alcoholism or Alzheimer’s disease)^71, 78–83^ or due to natural causes such as aging^84^. We think that the scientific community has reached a point where the numerous psychophysics reports of low-level perceptual losses in different pathologies that have appeared in the last decade are explainable with the accumulated knowledge from neurophysiology and computational modelling. Importantly, it is possible to model from the behavioural data the underlying network modifications that occurred in a given dysfunctional visual system, and thus should bring fundamental insights into multiple pathologies, their causes and effects, and hopefully new treatments.

## Materials and Methods

### Part I Psychophysics Experiments in Humans

#### Subjects

Fourteen naturally occurring anisometropic amblyopes 21 to 29 (24.7 ± 2.1) years (three females, Table 2), diagnosed by two ophthalmologists (two of the authors), and eleven neurotypical adults (including one of the authors) of similar age 24 to 41 years (28.4 ± 5.0, 5 females), naive to the purpose of the experiment (except the author), participated in the full study (low and high SF measures). Three amblyope’s data (in Table 2, subjects 12, 13, 14) were excluded in the analyses and results because the sensitivity values at high SFs of 2 AEs were null (could not see the stimuli at high SF, and thus they couldn’t go on with the tilt repulsion measure at high SF) and the third amblyope turned out to have had about 10 years of strong strabismic period during childhood (discovered in a post-measurement debriefing talk). The research protocol was approved by the Ethics Committee of the University of Science and Technology of China and followed the guidelines of the Declaration of Helsinki. Written informed consent was obtained from each participant after explanation of the nature and possible consequences of the study and they were paid for their participation on an hour basis. An additional batch of 8 anisometropic amblyopes, of similar age (22 to 29 years; 23.9 ± 0.8), were measured at low SF, as described in the main text, and their characteristics are presented in Supplementary Table S4. They were also diagnosed by two ophthalmologists (two of the authors), and naive to the purpose of the experiment.

**Table 2:** Characteristic of each amblyopic participant.

Subject	Sex	Age	Visual Acuity		Refractive Correction	
			Left eye	Right eye	Left eye	Right eye
1	M	26	0.8	0.4	−0.50DC × 165	+2.00DS/+3.00DC × 70
2	M	26	1	0.5	plano	+1.00DS/+1.50DC × 95
3	M	23	0.4	1.2	+2.50DS/1.50DC × 85	plano
4	M	24	0.5	1	−1.00DS/+3.50DC × 85	−2.00DS/−1.00DC × 10
5	M	23	1.5	0.15	plano	+4.00DS/+1.00DC × 85
6	F	24	0.25	1	+0.50DS/+1.00DC × 170	plano
7	F	26	0.8	0.2	−4.25DS/−0.50 × 15	−3.00DS/−1.00DC × 170
8	F	29	0.5	0.8	0.00DS/+0.50DC × 65	−2.75DS/−1.50DC × 20
9	M	21	1.2	0.5	−0.75DS	+2.50DS/+1.00DC × 170
10	M	25	0.8	0.6	Plano	+6.00DS/+1.00DC × 25
11	M	25	0.6	0.8	−1.75DS	+0.50DS/+0.50DC × 15
12	M	26	1	0.2	−1.00DS/−0.50DC × 160	+1.50DS/+1.50DC × 60
13	M	25	0.8	0.2	−2.25DS	+3.00DS/+1.50DC × 75
14	M	28	1.2	0.3	Plano	−3.00DS

#### Apparatus

All stimuli were displayed on a Sony MultiScan G520 monitor driven by an NVIDIA Quadro K600 video card and generated by a PC computer running Matlab (The Mathworks Corp., Natick, MA) with PsychToolBox 3 extensions^85, 86^. The monitor had a total display area of 40.0 cm × 30.0 cm, with a resolution of 1920 × 1440 pixels (1600 × 1200 pixels for 8 additional anisometropic amblyopes) and a refresh rate of 85 Hz. Participants with their best refractive corrections viewed the stimuli monocularly, which were presented on the centre of the monitor. The untested eye was occluded with an opaque eye patch (see Fig. 1a). A chin-rest equipped with a forehead strap was used to minimize subjects’ head movements during the experiment. Participants were seated in a darkened room in which all local cues to vertical/horizontal were removed by using black cardboard in front of the monitor to provide a circular window of 30.0 cm in diameter to the display^51^. The original 8 bits per pixel luminance range digitization was extended above 10 bits with the contrast box switcher^87^, providing the necessary minimum contrast steps for contrast detection measures, and the monitor weekly calibrated with a custom laboratory automated procedure.

#### Stimuli

For contrast sensitivity functions (CSF, representing the inverse of the minimum detectable contrast at various SFs) measure, stimuli were circular vertical sine-wave gratings of two degrees in diameter (Fig. 1c), presented on the display with 40 cd/m^2^ background luminance, with randomized phases across trials. To minimize edge effects, a border-mask was used to blend the stimuli into the background.

For tilt repulsion measure, stimuli (Fig. 1d) were a centre Gabor patch surrounded by a sine-wave grating seen through an annular window whose width was equal to the centre window diameter, presented on a mean luminance of 35 cd/m^2^. Stimulus size was scaled with the SF such that all stimuli had the same number of wavelengths within its window of visibility and we kept the centre window diameter of the target stimulus fixed at 4 cycles. The Gabor patch was defined through the following equation:

1 $${\rm{L}}(x,y)={L}_{0}+{L}_{0}C\times {e}^{-\frac{{x}^{2}+{y}^{2}}{{\sigma }^{2}}}\times \,\cos (2\pi f(x\,\cos \,\theta +y\,\sin \,\theta ))$$

with *L*_0_ the background luminance of the screen, *C* the Gabor patch contrast, and *f* and *θ* its SF and angle relative to vertical. The orientations of the surround grating were 0°, ±15°, ±30°, ±75° relative to the centre target, and both the contrast of the centre and surround was fixed at 90%. For both centre and surround, the cosine had a phase of zero.

#### Procedures

Firstly, VA was measured using a standard wall-mounted Tumbling E chart (Fig. 1b), from a distance of 5 metres. Then, each subject performed both contrast sensitivity and tilt illusion measurement. During the measurement, the observers were instructed to fixate a small black square displayed at the centre of the screen and that the stimuli would be briefly presented centred on it. They started each trial by pressing a keyboard button. Before each formal measurement, they were allowed a 50–100 trials training to each task. Breaks were set-up every 200 trials to prevent excessive fatigue. Psychometric curves for contrast detection (CSF) and orientation discrimination (tilt repulsion) were measured using the weighted up-down adaptive procedure^88^. CSF measures were done before tilt repulsion measures, in order to extract the peak SF as the low-SF and obtain a high SF individually for each subject. Tilt repulsion were measured in two separate blocks for each eye, one block for low SF, the other for high SF. High SF blocks were measured first.

*Contrast Sensitivity Measure*. The subjects’ monocular contrast sensitivity was measured under eleven SFs (0.71, 1, 1.41, 2, 2.83, 4, 5.66, 8, 11.31, 16, 22.63 cycles per degree, c/d (cycles per degree)). A viewing distance of 4 meters was used in CSF tests for all subjects. We measured each eye of the amblyopes in a random order, and randomly chose one eye for the control group (neurotypical adults). There were 165 trials in each measure (15 trials/SF). In each trial, 150 ms after the fixation disappeared, two intervals of 100 ms separated by a 500 ms inter-stimulus interval would be demarcated by a brief tone at the beginning of each interval. The signal sine-wave grating appeared in only one of the two intervals.

We used a 3-key response design^89^, with two keys for responding in which interval the subjects perceived the signal and a third key in the event they were undecided. Correct responses were accompanied with a high frequency sound (1 kHz) as a response feedback, and incorrect with a low frequency (0.5 kHz). Undecided key presses had no sound feedback. Signal contrast was varied according to the up-down staircase procedures as follows. Correct/incorrect responses were followed by a decrease/increase of contrast with steps 4.5/8 times the base 10% contrast (in log-units) for SFs of 0.71, 1.41, 2.83, 5.66, 11.31, 22.63 cycles per degree and for the remaining SFs the steps were 1.5/7 times the base 10% contrast (in log-units). Undecided key presses were randomly drawn as correct/incorrect. Starting points were contrasts of 0.5, 0.005, 0.5, 0.005, 0.5, 0.005, 0.5, 0.005, 0.7 0.05, 0.8 for the 11 successive SFs, respectively, and each staircase “down” step-size was additionally 3 times bigger for the first 4 trials.

*Tilt Repulsion Measure*. Monocular tilt repulsion effects were measured for each eye where CSF was measured. 200 ms after fixation point disappearance, the stimulus was presented for 9 frames (~100 ms). There was no feedback. Each eye was measured at two SFs (chosen from the CSF measure): one SF (called low-SF) was chosen near the optimal SF (at which subject’s contrast sensitivity is the highest), the second high-SF was higher and chosen to avoid being too close to the cut-off SF above which the subject cannot see full contrast stimuli. The viewing distance was 4 m for high-SF and 2 m for low-SF. Each block consisted of 420 trials (7 orientations × 2 × 30 trials) for all subjects.

We used a 3-key design for this measure as follows: the subject had to decide whether the centre target orientation was clockwise (CW) or anti-clockwise (CCW) from his/her internal vertical standard by responding on two predefined keys (for example, in Fig. 1d, the centre target is 15 degrees, the surround is 30 degrees to the target. In this case, the target is CW to the vertical); if the subject did not see the target (due to surround suppression; especially at high SFs, see results), he/she had to press the third key for target “not seen”. In this way, our experimental design allowed to exclude any plausible crowding effect and provided an indirect measure of surround suppression. Additionally, it is known that in anisometropic amblyopia this crowding effect seems to be similar to the neuro-typical person’s crowding, and any differences were small enough to be ignored in such cases^21, 38, 42^. For each SO, we sampled the psychometric functions by varying target orientation according to the up-down staircase procedure with steps Up/Down of 2/5 and 5/2 degrees corresponding to convergence points of about 71% and 29% for CCW/CW responses, and “not seen” cases were randomly drawn as CCW/CW. Staircases started at the opposite side of the convergence point allowing rapid measures within the transition region of the psychometric function.

#### Data Analyses

Bayesian fitting^90^ was used to adjust theoretical psychometric functions to the CSF and tilt discrimination data.

*Contrast Sensitivity Function*. A 2D psychometric function was fit to the 2D contrast-SF (*c*, *f*) data, with the percentage of correct response defined as:

2 $${\rm{P}}(c,f)=\gamma +\frac{1-\gamma -\lambda }{1+{e}^{-\mathrm{log}(\frac{21}{4})(\mathrm{log}(c)-\mathrm{log}(\frac{1}{S(f)}))/\sigma }}$$

with parameters γ and λ being subject’s “guess rate” (see below) and lapsing rate, and 2σ defining the spread between 16–84% of the function in the range γ to 1 − λ (assuming constant spread across SFs). *S(f)* is the standard 3-parameters sensitivity function^91, 92^:

3 $${\rm{S}}(f)=M{f}^{a}{e}^{-\frac{f}{b}}$$

used in previous studies to define the CSF shape in the SF dimension. The 3 response keys design data was processed following Garcia-Perez^93^, which in the event the subject followed the 3^rd^ key instructions allows a decrease in measurement variability. The lapsing rate was fixed at 1%. The “guess rate” γ was obtained for each CSF measure from the total proportion *p*_3_ of 3^rd^ key presses, by noticing that no 3^rd^ key presses lead to subject’s guess rate being γ = 50% (standard 2AFC, subjects produce their own guesses) and higher usage leading to lower than 50% guess rates. From simulation analyses, we fixed γ = max(0, (1 − *p*_3_/0.7)/2). The mean guess key usage across our subjects was 0.49 ± 0.21 (S.D.). Example CSF fits for one subject are displayed on Fig. 2, which displays the responses given by the subject to each presented stimulus.

*Tilt Repulsion*. We fit a 1D psychometric function to the orientation discrimination data for each SO, with probability of CW responses to target orientation *θ* given by:

4 $${\rm{P}}(\theta )=\lambda +\frac{1-2\lambda }{1+{e}^{-\mathrm{log}(\frac{21}{4})(\theta -a)/\sigma }}$$

where λ is subject’s lapsing rate, and *a* and *σ* being the perceived vertical orientation (also called “bias”) for the given surround and the threshold of the subject for perceiving a deviation from verticality, respectively. Because of the symmetry in the experimental design (symmetric surrounds of ±15, ±30, ±75 degrees), for the fitting we imposed that thresholds of opposite SOs (eg −30 and +30 degrees) are the same. The lapsing rate was fixed at 1% for all but one subject, where it was fixed at 0%. The data was processed by eliminating any datum with 3^rd^ key responses (subject did not see the target), thus providing a psychometric data for clear orientation perception, and we computed the amount of surround suppression as the proportion of 3^rd^ key presses. Bias values were computed as the half-difference between two opposite SOs. Proportions were also pooled for opposite SOs.

#### Statistical Analysis

We performed 3-way within-subject ANOVA to compare AE to NAE within the same subjects for SOs and SFs, and between-within subject ANOVA to compare neuro-typical eye (NTE) to AE or NAE. The parallel surround configuration had a strong detrimental effect on the detectability of the centre target despite its nearly full contrast. Therefore, it automatically decreased the number of trials available for the correct estimation of orientation perception, especially at high SFs, for the iso-orientation configuration. And at SOs of 75, it was the indirect tilt effect, widespread to be processed in the higher visual cortex^48^, which we won’t discuss further. As a consequence, we restricted our data analyses to SOs of 15, 30 corresponding to the direct tilt repulsion effect. All statistical levels use Geisser-Greenhouse epsilon-hat adjusted values, where appropriate. The CSF fits provided us with an estimate of subjects’ sensitivity across all SFs. In the present report we restricted our presentation to the two low- and high-SF sensitivities chosen during the measures as they are relevant to the tilt measurements. The tilt measures provided us data simultaneously about surround suppression effects (proportions of not seen) as well as tilt repulsion effects of SO onto the centre target (biases). Each of these variables were analysed through the ANOVA. Correlation analyses were based on Spearman’s rank correlation. p < 0.05 is considered significant. Independent Sample t-test used *df*-adjustment when the variances were not equal, which was checked with Levene’s test for equality of variances.

### Part II Computational Modelling Of V1

#### Neurophysiological Accounts of V1 Neurons Tuning Relations

The simple model of V1 cells we tested in the study is based on multiple tuning characteristics of each cell: orientation, SF and contrast. These tuning functions are characterized very well among different animal species, and it was found that the parameters between or within these tunings co-vary. Since our model uses these properties, we had to fix the relations between the characteristic parameters, which we did based on the various physiological reports, summarized here.

About the contrast tuning, the CRF was chosen as the usual hyperbolic ratio function (equation (9) in model description)^94^. In this equation, the most important variable for our model is the semi-saturation constant *c*_*k*_, normally representing the contrast at which the cell reaches half of its maximum amplitude. It was found that *c*_*k*_ and power *n* co-vary across the neuronal population^60, 95^. First, since we need the relation only between the neurons with lowest *c*_*k*_ (best sensitivity at a given SF), we fixed *n* = 2 for all neurons. Second, neuronal sensitivity was found to vary with the preferred SF of the neuron^30, 94, 96^. For the purpose of CSF modelling, we found that the envelope of the population of neuronal sensitivities varies with the SF in a similar way as the CSF theoretical equation (equation (3)) (the bell-shaped upper boundary of the neurophysiological data is well described by the standard CSF equation; data extracted from Kiorpes *et al*., 1998 and fit not shown). Therefore, we also used equation (3) to describe the neuronal sensitivity function in the model (equation (25); Fig. 5e). Last, the standard hyperbolic equation used in the literature has the disadvantage that for low powers of *n* or increasing *c*_*k*_, the semi-saturation constant *c*_*k*_ does not represent the contrast at half-maximum any more. Thus, one has to recompute the half-amplitude constant, *c*_*½*_, as:

5 $${c}_{1/2}^{n}=\frac{{c}_{k}^{n}}{1+2{c}_{k}^{n}}$$

This parameter is also reported in Fig. 5e, and g with the arrows. We fixed the SF tuning width *σ*_*SF*_ to decrease with higher preferred SFs *f*_*j*_ (equation (26)), as reported physiologically by various authors^97–99^.

#### Simple Model of V1 Surround-to-Centre Interactions

We assume, as in many previous studies, that simple feature perception as local orientation and contrast can be explained through the decoding of primary visual cortex neuronal activities. Therefore, we investigated a simple V1 model of two-layer neurons coding the main features of interest in the study: orientation, contrast, SF, and space. The model consists of orientation hyper-columns arranged into a hexagonal structure, with each hyper-column containing neurons responding to various contrasts and SFs. First layer neurons can be thought of simple cells whose responses are as follows:

6 $${r}_{ijk}(\theta ,f,c)=A\times T(\theta ;{\theta }_{i})\times F(f;{f}_{j})\times C(c;{c}_{k})$$

with “preferred” features (*θ*_*i*_, *f*_*j*_, *c*_*k*_) and the three normalized tuning functions to orientation, SF and contrast are described as wrapped-Gaussian^100^, log-Gaussian^101^ and hyperbolic ratio^94^, respectively (*A* is the maximum amplitude of firing of the neuron). They are:

7 $$T(\theta ;{\theta }_{i})={e}^{-\frac{1}{2}{(\frac{\theta -{\theta }_{i}}{{\sigma }_{\theta }})}^{2}}$$

8 $$F(f;{f}_{j})={e}^{-\frac{1}{2}{(\frac{{\mathrm{log}}_{2}f-{\mathrm{log}}_{2}{f}_{j}}{{\sigma }_{SF}})}^{2}}$$

9 $$C(c;{c}_{k})=\frac{{c}^{n}}{{c}_{k}^{n}+{c}^{n}}$$

Remark: for the contrast tuning, ck is the semi-saturation constant and can be called the “preferred” contrast of the neuron, since for contrasts around ck the neuron is the most informative above the input contrast^57, 58, 60^, and away from ck it asymptotes and provides no information about contrast input.

These simple cells feed the second layer of neurons through a spatial (excitatory centre)–(inhibitory surround) connectivity structure, whose responses *R*_*ijk*_ follow the conductance-based model^53, 102^:

10 $${R}_{ijk}(\theta ,f,c)=h({v}_{ijk})$$

11 $$\tau \frac{{\rm{d}}{v}_{ijk}}{dt}=-{v}_{ijk}+({v}_{e}-{v}_{ijk}){g}_{e}+({v}_{i}-{v}_{ijk}){g}_{i}$$

12 $${g}_{e}=\sum _{mno}{\omega }_{ijk,mno}^{cen}{r}_{mno}$$

13 $${g}_{i}=\sum _{mno,(x,y)}{\omega }_{ijk,mno,(x,y)}^{sur}{r}_{mno,(x,y)}$$

with *h(*) a transducer (rectifying) function transforming voltage to firing rate and feature weights *ω*’s defined as:

14 $${\rm{h}}(v)=m\times max\,(0,v-T)$$

15 $${\omega }_{ijk,mno}^{cen}={I}_{c}{G}_{im}^{c}{G}_{jn}^{c}$$

16 $${\omega }_{ijk,mno,(x,y)}^{sur}={I}_{s}{G}_{im}^{s}{G}_{jn}^{s}{G}_{x,y}$$

and the various parameters are: *T* is the voltage threshold of firing, *m* is the slope of voltage-to-firing rate relation, *τ* is the cell time constant, *v*_*e*_ and *v*_*i*_ are the excitatory and inhibitory equilibrium voltage potentials, *g*_*e*_ and *g*_*i*_ are respectively the excitatory and inhibitory conductances feeding the corresponding neuron through a weighted sum of first layer activities (*g*_*e*_ sum is within hyper-column; *g*_*i*_ sum is over all surrounding hyper-columns), *G*_*im*,*jn*_ are Gaussian tuned feature weights (respectively within orientation and within SF; with possible different tuning widths indexed (*c*, *s*)), *G*_*x*,*y*_ is a spatial weight function summing surrounding hyper-columns activity, and *I*_*c*,*s*_ are the centre/surround excitatory/inhibitory input strengths, respectively. Here, it is assumed that the weights are independent across features and iso-feature tuned (peaking at the receiving neuron preferred value (*i*, *j*), i.e., iso-orientation and iso-SF).

In the feed-forward model equation (10) can be analytically solved, giving:

17 $${v}_{ijk}=\frac{{v}_{e}\,{g}_{e}+{v}_{i}\,{g}_{i}}{1+{g}_{e}+{g}_{i}}$$

18 $${R}_{ijk}=h({v}_{ijk})$$

Using all relations above and an input with uniform surround (all surrounding hyper-columns are stimulated with the same stimulus of orientation *θ*_*s*_ and contrast *c*_*s*_), assuming the centre stimulus (*θ*_*c*_, *c*_*c*_) excites the centre hyper-column, the centre input excitatory/inhibitory conductances can be analytically computed:

19 $${g}_{e,i}=\frac{{I}_{c,s}{A}_{c,s}}{{\sigma }_{\theta }^{c,s}{\sigma }_{SF}^{c,s}}\sum _{\theta }^{c,s}\,{B}_{C,S}\times {e}^{-\frac{1}{2}\frac{{({\theta }_{i}-{\theta }_{c,s})}^{2}}{{({\sigma }_{\theta }^{c,s})}^{2}+{\sigma }_{\theta }^{2}}}\times {e}^{-\frac{1}{2}\frac{{({\mathrm{log}}_{2}f-{\mathrm{log}}_{2}{f}_{j})}^{2}}{{({\sigma }_{SF}^{c,s})}^{2}+{\sigma }_{SF}^{2}}}$$

The various constants are defined as:

20 $${B}_{C,S}=\sum _{SF}^{c,s}{e}^{(\frac{{({{\sum }^{}}_{SF}^{c,s})}^{2}{(\mathrm{log}(2))}^{2}}{2}+{K}_{c,s}\mathrm{log}(2))}$$

21 $${({{\sum }^{}}_{\theta }^{c,s})}^{-2}={({\sigma }_{\theta }^{c,s})}^{-2}+{({\sigma }_{\theta })}^{-2}$$

22 $${({{\sum }^{}}_{SF}^{c,s})}^{-2}={({\sigma }_{SF}^{c,s})}^{-2}+{({\sigma }_{SF})}^{-2}$$

23 $${K}_{c,s}(f;{f}_{j})=\frac{{({\sigma }_{SF}^{c,s})}^{2}{\mathrm{log}}_{2}f+{\sigma }_{SF}^{2}{\mathrm{log}}_{2}{f}_{j}}{{({\sigma }_{SF}^{c,s})}^{2}+{\sigma }_{SF}^{2}}$$

where ${\sigma }_{\theta }^{c,s}$ and ${\sigma }_{SF}^{c,s}$ are the orientation and SF tuning widths of the weight functions *G*_*im*_ and *G*_*jn*_ for centre-centre and surround-to-centre connections, respectively, *A*_*c*,*s*_ are the contrast-weighted amplitude of firing of the input neurons *r*_*ijk*_ for centre/surround respectively, *I*_*c*,*s*_ are the excitatory and inhibitory weight amplitudes, and

24 $${I}_{s}={n}_{s}\times {I}_{inh}$$

defines the total inhibitory input from all surrounding hyper-columns with mean inhibitory strength per hyper-column *I*_*inh*_, respectively; *n*_*s*_ is a “mean” number of surround hyper-columns influencing the centre.

#### Fixed Parameters

Summary of the set of parameters that were fixed in the model (following a “normalized” conductance based subtractive inhibition model e.g., ref. 53): *n*_*s*_ = 6, *n* = 2, *A* = 2, *σ*_*SF*_ = 1, *m* = 1, *T* = 1, *I*_*c*_ = 1, *v*_*e*_ = 14/3, *v*_*i*_ = −2/3. SF dimension sampling was every ¼ octaves from ½ up to 64 c/d, orientation feature sampling every 2 degrees. The contrast tuning relation was kept normalized by multiplying its amplitude by a factor *(1* + *c*_*k*_^*n*^ *)*.

#### Model Based Fitting of CSF and Tilt Perception Data

Here we investigate how based on the output activity *R*_*ijk*_ of the network we can predict the perception of the subject in the two main features of interest, contrast detection for predicting the contrast sensitivity function and orientation identification for predicting the tilt repulsion effect. It is assumed that perception is based on decoding of the centre hyper-column activities as described below.

*Modelling the Contrast Sensitivity Function (CSF in 2D)*. In this experiment, the target stimulus is a vertical and uniform sine-wave grating limited in a circular spatial window, whose strength (contrast) is varied in order to measure the perception threshold across all SFs.

Given the uniform input stimulus, all neurons *r*_*ijk*_ have exactly the same input and thus their activation across orientation, SF, and contrast have the same profiles and peaks (for any *i*, *j*, *k*: ${r}_{ijk}^{c}={r}_{ijk}^{s}$). Second, given the task of detecting always a vertically oriented grating, by assuming that subjects disregard other orientated activities through an unspecified attentional mechanism, we simplified the term over orientations in equation (19) into a value of one (in practice, this simplification can be thought of pooling these orientation neuronal activities into the constants *I*_*c*,*s*_ or *A*_*c*,*s*_) and modelled only one orientation of network activities.

In the above model description, one important function in predicting the contrast sensitivity at a given SF is the hyperbolic ratio of the neuronal population^57, 58, 60^. We assume that contrast detection across all SFs is performed by decoding the activities of the neurons with the smallest semi-saturation constant ${c}_{k}^{min}(SF)=min({c}_{k}|SF)$. In our model, to predict the CSF across SFs we additionally need to properly describe the relation between ${c}_{k}^{min}$ and SF together with SF tuning width versus preferred SF. Based on previous neurophysiological reports, we fixed:

25 $${c}_{k}^{min}({f}_{j})={c}_{min}\times \frac{{(ab)}^{a}{e}^{-a}}{{f}_{j}^{a}{e}^{-\frac{{f}_{j}}{b}}}$$

26 $${\sigma }_{SF}={e}^{-\frac{{f}_{j}-2}{0.2}}$$

where *f*_*j*_ is the preferred SF of the neuron and parameters (*c*_*min*_, *a*, *b*) define the neuronal sensitivity function across SFs. We fixed ${\sigma }_{SF}^{c,s}={\sigma }_{SF}/2$ because of centre/surround tuning widths entanglement in predicting behavioural results, *I*_*c*_ = 1; *n*_*s*_ = 6, and neurons with *c*_*k*_ > 1000 were pruned.

Last step, for predicting the contrast sensitivity function across SFs, we used the standard signal detection theory, defining the psychometric function:

27 $${\rm{P}}(c,f)=\gamma +(1-\gamma -\lambda ){P}_{th}$$

28 $${P}_{th}=2({\int }_{0}^{+\infty }\frac{{R}_{j}-{R}_{0}}{\sqrt{Var({R}_{j})+Var({R}_{0})}}d{R}_{j}-0.5)$$

with the lapse and guess rates obtained as described in the above section *Data analyses*, and *R*_0_ being the activity of the neurons for no signal input.

From the above model description, there are only four free model parameters that need to be adjusted for predicting the full CSF: *c*_*min*_, *I*_*inh*_, *a*, *b*. It was done by replacing equation (2) with equation (27) and following all remaining steps.

*Modelling Orientation Identification (Tilt Illusion)*. For this feature, we make a different set of simplifications in the model feature space based on the experimental design for tilt perception. Here, centre and surround hyper-columns are stimulated with varying orientations while the contrast of the centre and surround stimuli are kept constant and equal. Therefore, we can describe the two-layer neuronal network activities through the above mathematical development. But here, we fixed the input layer contrast activation at *A* = 2 and *c* = 1 (near maximum contrast eg., ref. 53), and given the task of identifying the orientation of the centre stimulus for a fixed SF^45^, we simplified into equation (19) the term over SFs by assuming subjects disregard other SF neuronal activities (eg through an unspecified attentional mechanism), giving:

29 $${g}_{e,i}=\frac{{I}_{c,s}{A}_{c,s}}{{\sigma }_{\theta }^{c,s}}\sum _{\theta }^{c,s}({e}^{-\frac{1}{2}\frac{{({\theta }_{i}-{\theta }_{c,s})}^{2}}{{({\sigma }_{\theta }^{c,s})}^{2}+{\sigma }_{\theta }^{2}}})$$

with all constants defined in model description section. We fixed the centre and surround feed-forward orientation tuning widths to ${\sigma }_{\theta }^{c,s}={\sigma }_{\theta }/2$.

Last, to predict the orientation psychometric function (tilt data), we decoded the perceived orientation of the stimulus (*a* in equation (4)) as the vector average orientation of the centre hyper-column activities^44, 51, 103^. For this feature, there are only two free parameters, *I*_*inh*_ and *σ*_*θ*_, that are sufficient to provide tilt misperception description^44, 51^. Lapse rate was fixed at 1%.

#### General Fitting Procedure

First, each individual data set was fit with the tilt prediction part only for SO of ±15 and ±30 degrees in order to extract *I*_*inh*_ at both high and low SFs. For a given SF, a single discrimination threshold for all SOs was set as free parameter. Then, the parameter *I*_*inh*_ was used as fixed in the CSF fitting procedure in order to find the best three parameters (*c*_*min*_, *a*, *b*) that described subject’s CSF data.

#### Definition of Vector Blur

Vector blur^2^, which relates to the optics of the eye, is defined as follows:

30 $$Vector\,blur=\frac{\sqrt{{s}^{2}+sc+{c}^{2}}}{2}$$

where *s* represents the spherical refractive error and *c* represents the cylindrical refractive error.

### Data Availability

The datasets generated during and/or analysed during the current study are available from the corresponding author on reasonable request.

## Electronic supplementary material

Supplementary Information
