# Supplementary material for: The neural basis of spatial vision losses in the dysfunctional visual system
Source: Sci Rep. 2017 Sep 12;7:11376. doi: 10.1038/s41598-017-11364-0 (PMC5595843; doi:10.1038/s41598-017-11364-0)
Supplement: Supplementary file 1 — Supplementary Information [file 41598_2017_11364_MOESM1_ESM.pdf]

Supplementary Information to

The neural basis of spatial vision losses in the dysfunctional visual system

Jinfeng Huang[1], Yifeng Zhou[1,*], Caiyuan Liu[2], Zhongjian Liu[2], Chunmeng Luan[3] Tzvetomir Tzvetanov[1,*4]

[1] Chinese Academy of Sciences Key Laboratory of Brain Function and Disease, and School of Life Sciences, University of Science and Technology of China, Hefei, Anhui, People's Republic of China;

[2] Research and Treatment Center of Amblyopia and Strabismus, University of Science and Technology of China, Hefei, Anhui, People's Republic of China

[3] Technical Research & Development Center, North Huajin Chemical Industries Group Corporation, Panjin, Liaoning, People's Republic of China

[4] School of Computer & Information, Hefei University of Technology, Hefei, Anhui 230009, People's Republic of China

Supplemental Figures

Supplemental Tables

Supplementary Material

Supplementary Figure legends

Supplementary Figure S1 | Sensitivity vs. VA or Vector Blur of 21 amblyopic participants.

Sensitivity at low SF vs. decimal visual acuity (VA) (a) or vs. Vector blur (b). (r and p are Spearman rank correlations and probability; $p<0.05$ is considered significant). ○ represents AE; □ represents NAE. Each symbol with a dot in the center is from the 10 additional subjects.

Supplementary Figure S2 | CSF and Tilt Illusion Amplitudes Co-vary in 21 Amblyopic Eyes.

(a-b) Correlation between tilt repulsion (bias) and contrast sensitivity at the lowest measured SF for surround orientations of (a) ±15 degrees and (b) ±30 degrees. ○ represents AE; □ represents NAE. Each symbol with a dot in the center is from the 10 additional subjects.

Supplementary Table Legends

Supplementary Table S1 | Analysis Results of Surround Suppression Effect.

Supplementary Table S2 | Analysis Results of Orientation Discrimination Thresholds.

Supplementary Table S3 | Analysis Results of Orientation Discrimination Perception.

Supplementary Table S4 | Characteristic of 8 new amblyopic participants.

Supplementary Table S5 | Correlation results between model parameters for each eye type

Supplementary Table S6 | Analysis of Variance results of Two groups of amblyopes on tilt repulsion measure.

Supplementary Figures

Supplementary Figure S1 | Sensitivity vs. VA or Vector Blur of 21 amblyopic participants.

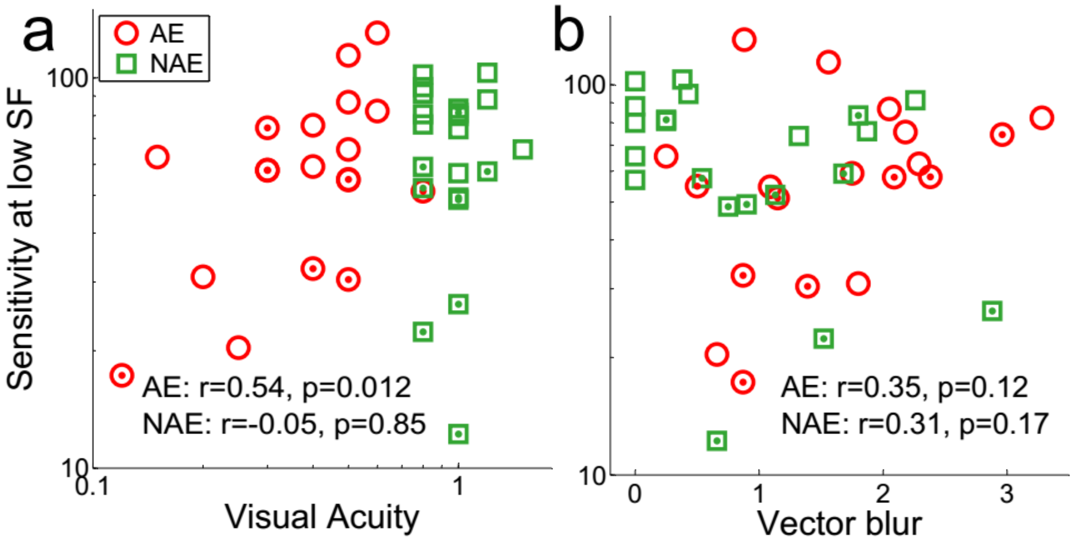

Supplementary Figure S2 | CSF and Tilt Illusion Amplitudes Co-vary in 21 Amblyopic Eyes.

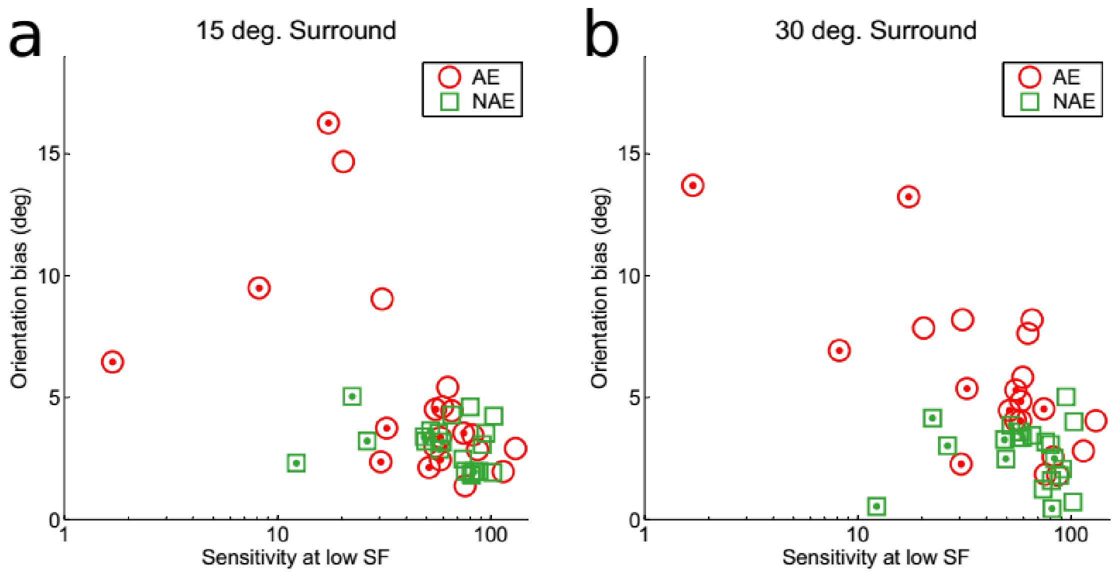

Supplemental Tables

Supplementary Table S1 | Analysis Results of Surround Suppression Effect.

	Factors	F value	P value
AE vs. NAE	Eye	$F(1,10)=3.7$	0.085
	SO	$F(3,30)=17.7$	0.000011
	SF	$F(1,10)=11.4$	0.0071
	SO×SF	$F(3,30)=18.4$	0.000024
AE vs. NTE	Eye	$F(1,20)=0.28$	0.60
	SO	$F(3,60)=25.2$	$2.6*10^{-8}$
	SF	$F(1,20)=25.1$	$6.6*10^{-5}$
	SO×SF	$F(3,60)=21.1$	$2.6*10^{-7}$
NAE vs. NTE	Eye	$F(1,20)=0.03$	0.86
	SO	$F(3,60)=27.2$	$1.5*10^{-7}$
	SF	$F(1,20)=27.8$	$3.6*10^{-5}$
	SO×SF	$F(3,60)=22.2$	$3.2*10^{-7}$

Supplementary Table S2 | Analysis Results of Orientation Discrimination Thresholds.

	Factors	F value	P value
AE vs. NAE	Eye	F(1,10)=0.013	0.91
	SO	F(1,10)=8.513	0.015
	SF	F(1,10)=18.6	0.0015
	SO×SF	F(1,10)=9.5	0.012
AE vs. NTE	Eye	F(1,20)=7.48	0.0128
	SO	F(1,20)=8.08	0.0101
	SF	F(1,20)=67.7	$7.5*10^{-8}$
	SO×SF	F(1,20)=8.2	0.0097
NAE vs. NTE	Eye	F(1,20)=5.2	0.034
	SO	F(1,20)=6.46	0.019
	SF	F(1,20)=15.1	0.0009
	SO×SF	F(1,20)=5.5	0.029

Supplementary Table S3 | Analysis Results of Orientation Discrimination Perception.

	Factors	F value	P value
AE vs. NAE	Eye	F(1,10)=3.87	0.077
	SO	F(1,10)=40.4	0.0001
	SF	F(1,10)=107.4	$1.1*10^{-6}$
	SO×SF	F(1,10)=36.2	0.0001
AE vs. NTE	Eye	F(1,20)=8.997	0.0071
	SO	F(1,20)=24.9	0.00007
	SF	F(1,20)=84.7	$1.2*10^{-8}$
	SO×SF	F(1,20)=32.5	$1.4*10^{-5}$
NAE vs. NTE	Eye	F(1,20)=4.57	0.045
	SO	F(1,20)=58.3	$2.4*10^{-7}$
	SF	F(1,20)=168.4	$3.4*10^{-11}$
	SO×SF	F(1,20)=40.7	$3.2*10^{-6}$

Supplementary Table S4 | Characteristic of 8 new amblyopic participants.

Subject	Sex	Age	Visual Acuity		Refractive Correction	
			Left eye	Right eye	Left eye	Right eye
1	M	24	0.5	1.0	-1.00DS	-1.50DS
2	M	23	0.8	1.0	+2.00DS /+0.50DC*135	-5.75DS
3	M	22	0.12	0.8	+1.00DS/+1.00DC*95	-2.75 DS/-0.50DC*10
4	M	24	1.2	0.4	-0.75DS/-0.50DC*175	+1.00DS/+1.00DC*85
5	M	24	0.3	1.0	+5.50DS/0.75DC*95	+3.00DS/+1.00DC*85
6	M	22	0.3	0.8	+3.75DS/0.75DC*115	-2.75DS/-1.00DC*20
7	M	23	0.3	1.0	+4.50DS/0.50DC*35	-1.50DS/-0.50DC*30
8	M	29	0.5	1.0	+2.50DS/0.50DC*90	-0.50DS

Supplementary Table S5 | Correlation results between model parameters for each eye type

Correlated Parameters	AE	NAE	NTE
c_{min} vs. I_{inh}	r=-0.19<br>p=0.399	r =0.24<br>p =0.285	r =0.05<br>p =0.881
σ_θ vs. I_{inh}	r =-0.37<br>p =0.103	r =-0.46<br>p =0.037	r =-0.20<br>p =0.558
σ_θ vs. c_{min}	r =-0.32<br>p =0.158	r =0.13<br>p =0.566	r =-0.05<br>p =0.881

Supplementary Table S6 | Analysis of Variance results of Two groups of amblyopes on tilt repulsion measure.

	Factors	F value	P value
Surround Suppression Effect	Eye	F(1,19)=2.844	0.108
	SO	F(1,19)=1.280	0.272
	SO ×Eye	F(1,19)=0.388	0.541
	Group	F(1,19)=1.193	0.288
Orientation Discrimination Thresholds	Eye	F(1,19)=9.376	0.006
	SO	F(1,19)=1.087	0.310
	SO ×Eye	F(1,19)=2.084	0.165
	Group	F(1,19)=2.181	0.156
Tilt Misperception	Eye	F(1,19)=13.4	0.002
	SO	F(1,19)=0.094	0.763
	SO ×Eye	F(1,19)=2.816	0.110
	Group	F(1,19)=0.360	0.556

Supplementary Material

Re-analysis on all amblyopic subjects' physical state and comparison between the two batches of subjects on TI data.

On the one hand, in ***supplementary fig. S1***, we present the visual acuity (VA) (a) and vector blur (b) for each eye versus the contrast sensitivity at a lower SF, respectively; the symbols with a dot in the center are from the 10 new additional subjects. From the results of correlation analysis, we can conclude that the physical differences of the eye were unrelated to anisometropic amblyopes' perceptual sensitivity changes (r and p values can be seen from the supplementary fig. S1). On the other hand, we checked whether the two anisometropic amblyopia groups have differences in tilt repulsion measure. We performed between-within subject ANOVAs to compare the first batch data to the second batch data at lower SF on the surround suppression effect, orientation discrimination thresholds and tilt misperception, separately. The comparison of each index considered the factor of surround orientation (15°, 30°) and eye (NAE, AE). The results show that the two groups of amblyopes have no differences (see ***Supplementary Table S6*** *for detail*). In summary, the 10 new additional subjects' data can be used in the further analysis.
